# Expressive writing to reduce depressive and anxiety symptoms among sexual minority veterans: Study design for a pilot randomized controlled trial

**DOI:** 10.1016/j.conctc.2026.101650

**Published:** 2026-05-27

**Authors:** Kelly L. Harper, Katherine Kelton, Nicholas A. Livingston, Katherine M. Iverson, Colleen A. Sloan, Abigail Batchelder, Brian P. Marx

**Affiliations:** a National Center for PTSD, Behavioral Science Division, VA Boston Healthcare System, Boston, MA, USA; b Psychiatry Department, Chobanian & Avedisian School of Medicine, Boston University, Boston, MA, USA; c VA Boston Healthcare System, Boston, MA, USA; d National Center for PTSD, Women's Health Sciences Division, VA Boston Healthcare System, Boston, MA, USA; e Boston University, Chobanian & Avedisian School of Medicine, Department of Psychiatry, Boston, MA, USA; f Boston Medical Center, Department of Psychiatry, Boston, MA, USA; g The Fenway Institute, Fenway Health, USA

**Keywords:** Veteran, Sexual minority, LGBQ+, Expressive writing, Minority stress

## Abstract

**Background:**

Sexual minority veterans (e.g., gay, lesbian, bisexual) are at increased risk for depression and anxiety due in part to the experience of sexual minority stressors. However, few interventions target distress related to sexual minority stressors, and even fewer have been tested in Veterans Health Administration (VHA). Brief, easy to implement interventions, such as expressive writing-based interventions, that target distress related to sexual minority stressors are needed to reduce disparities in depression and anxiety.

**Method:**

We describe the design of a two-phase study consisting of an open trial and a pilot randomized controlled trial (RCT) comparing Expressive Writing on Minority Stressors (EWMS) and a neutral writing condition among sexual minority veterans. The EWMS protocol will be iteratively refined in a case series (n = 10) and then tested in a pilot randomized control trial (RCT; n = 54). Aims of the pilot RCT are to: 1) demonstrate feasibility and acceptability of study procedures and EWMS using quantitative and qualitative data; and 2) examine preliminary trends in depressive and anxiety symptoms to inform the design of a future, fully powered efficacy trial. Participants will be assessed at baseline, posttreatment, and 3 months after treatment. Qualitative exit interviews will be conducted to inform refinements to the intervention with an eye towards usefulness, satisfaction, and implementation.

**Conclusions:**

This pilot RCT will inform the first fully powered clinical trial to test an individually delivered intervention for sexual minority veterans to reduce depressive and anxiety symptoms associated with minority stress.

## 1 Expressive writing to reduce depressive and anxiety symptoms among sexual minority veterans: study design for a pilot randomized controlled trial

Sexual minority veterans (i.e., veterans who identify as gay, lesbian, bisexual, queer or other minoritized sexual orientations) represent a high-risk population for psychiatric disorders. Compared with heterosexual veterans, sexual minority veterans are two times more likely to have depressive and alcohol use disorders, three times more likely to report lifetime suicidal ideation (SI) and have substance use disorders [1,2], and are more than twice as likely to die by suicide [3]. Moreover, compared with their non-veteran peers, sexual minority veterans have greater posttraumatic stress disorder (PTSD), depression, and anxiety symptom severity [4]. Thus, interventions that address contributing factors for the higher prevalence and severity of psychiatric disorders are urgently needed for members of this community.

Results from prior studies with a variety of samples [5,6], observational and experimental methods [7,8], and both cross sectional and longitudinal designs [5,9,10] have shown that exposure to minority stressors in part explains sexual minority individuals’ higher prevalence and severity of depression, anxiety, and other outcomes. Exposure to sexual minority stressors is associated with both universal stress reactions [5,11] and reactions specific to sexual minority lived experiences, such as identity concealment and expectations for rejection [5,12]. These stress responses contribute to increased risk of anxiety and depression, which in turn explain risk for high-risk health behaviors (e.g., substance use and suicide) [13]. Therefore, sexual minority stressors are critical targets for psychotherapeutic intervention.

However, few interventions specifically target distress related to sexual minority stressors, and most that exist are lengthy (i.e., 10 to 12 sessions) [14]. Interventions of this length tend to have higher attrition and are more difficult to implement due to limited trained staff and resources [15]. Additionally, most interventions targeting minority stress are offered in group formats leaving few options for individuals interested in one-on-one care [14,16,17]. Brief one-on-one interventions targeting distress related to sexual minority stressor exposure are needed for sexual minority veterans given their greater depression and anxiety prevalence and severity. Brief interventions are important because they can be disseminated broadly, are cost-effective, and can significantly improve sexual minority people's health [18]. They can also serve as an adjunct to existing treatment options for depression and anxiety and be implemented in settings with lower acuity, such as primary care.

## 2 Expressive writing-based intervention

Expressive writing is a technique in which an individual writes about their thoughts and emotions related to a stressful experience in response to structured writing prompts. Research over the past 40 years has consistently shown that expressive writing can improve mental and physical health [[18], [19], [20], [21], [22], [23], [24], [25], [26], [27], [28], [29]]. The standard expressive writing paradigm involves writing between 3 and 5 times in response to a structured prompt for up to 30 min per writing occasion. Both quantitative and qualitative data indicate that writing about emotions and cognitions related to sexual minority stressors for at least 3 writing sessions reduces depression and anxiety symptoms among non-veteran sexual minority people [18,22,[30], [31], [32], [33], [34]]. Expressive writing on sexual minority stressors has reduced perceived stressfulness of the minority stressor [30], negative mood [18,22,31], identity concealment [22], and improved perceptions of social support [22,32].

Although expressive writing on sexual minority stressors has not been tested with sexual minority veterans, an expressive writing-based intervention, Written Exposure Therapy (WET) for PTSD, effectively reduces PTSD and depression symptom severity among veterans [24,35]. WET is being disseminated across Veteran Health Administration (VHA) clinics [23] and has been implemented in a range of treatment settings (e.g., primary care, specialty mental health, inpatient psychiatry). Notably, significantly fewer people drop out of WET than people who have received other evidence-based PTSD treatments [35]. It also requires less training for providers compared with other treatments. Based on abundant research indicating that expressive writing interventions are beneficial for reducing depression and anxiety and have been successfully implemented in VHA, an expressive writing intervention focused on sexual minority stressors may be a promising intervention for addressing the deleterious impacts of sexual minority stressors among veterans.

This paper describes the protocol and study design to refine and pilot an expressive writing intervention for sexual minority veterans, Expressive Writing on Minority Stressors (EWMS). As expressive writing on sexual minority stressors has only been implemented with non-veterans, we plan to conduct a formative open-trial case series with 10 sexual minority veterans to iteratively refine and optimize the writing prompts and delivery of the intervention (Phase I) prior to testing in a pilot randomized controlled trial (RCT) (Phase II). The aim of the trial is to examine the feasibility and acceptability of both the study design and the intervention. The second aim of the trial is to describe trends in depressive and anxiety symptoms over time to inform the design of a future, fully powered efficacy trial.

## 3 Methods

### 3.1 Trial design

This pilot RCT will use a two-arm 1:1 parallel RCT design. The trial will take place at [redacted for review]. Participants will be randomly assigned to EWMS or a neutral writing condition with a 1:1 allocation using a computer generated randomization block design. A statistician not involved in the evaluation or execution of the trial will use R to generate the randomization allocation. Block sizes will not be known to the PI to ensure concealment. Randomization will be conducted by study staff that are not involved in delivery of treatment or assessment. The primary outcomes of the trial will be feasibility of the trial design (i.e., recruitment rates, retention rates) and intervention (i.e., percentage of treatment completion) and acceptability of the intervention (i.e., treatment satisfaction, helpfulness, and usefulness). All results related to depressive and anxiety symptom outcomes will be interpreted as preliminary trends as the purpose of the trial is to establish feasibility and acceptability of the trial design and EWMS to inform the design of a future, fully powered efficacy trial. Assessments will be completed at baseline, posttreatment, and 3-month post treatment via telephone diagnostic interviews and online self-report questionnaires. The trial has been reviewed and approved by the Institutional Review Board at VA Boston Healthcare System and is registered with ClinicalTrials.gov NCT05897021.

### 3.2 Participants

The total sample for the trial will include 64 sexual minority veterans who are at least 18 years of age. Phase I will include 10 sexual minority veterans and Phase II will include 54 sexual minority veterans. The inclusion criteria includes: (1) being a veteran (2) identifying as a sexual minority person (i.e., identifying as gay, lesbian, bisexual, pansexual, queer, or another identity other than heterosexual), (3) endorsing clinically significant depressive or anxiety symptoms (score above 10 on the Patient Health Questionnaire-9 [PHQ-9] [36] or the Generalized Anxiety Disorders-7 [GAD-7] [37]), (4) reporting a history of sexuality-based minority stressor exposure that is contributing to distress based on the endorsement of at least one item on the Everyday Discrimination Scale (EDS) [38] that is contributing to distress per veteran report (i.e., “Are you currently experiencing distress [e.g., sadness, anger, anxiety, etc.] related to any of the above [discrimination] experiences?”), and (5) if on psychotropic medication, being on a stable dose for at least 4 weeks. Exclusion criteria includes: (1) clear and current suicidal plan and/or intent (assessed via the Columbia Suicide Severity Rating Scale [C-SSRS] [39]), (2) current presentation of unstable mania and/or psychosis (assessed via the Structured Interview for DSM-5 [SCID]), (3) current substance use disorder, severe (assessed via the SCID [40]), and (4) significant cognitive impairment determined by an inability to comprehend baseline screening questionnaires.

### 3.3 Procedures

#### 3.3.1 Recruitment

Veterans will be recruited from local mental health clinics and primary care clinics at [masked] via referrals from clinicians or self-referred through fliers distributed throughout the hospital campuses. We will also recruit through local hospitals, veteran organizations, and LGBTQ + organizations via flyers, emails on listservs, and on social media to recruit veterans who may not already be receiving care at [masked].

#### 3.3.2 Screening

**Fig. 1:**
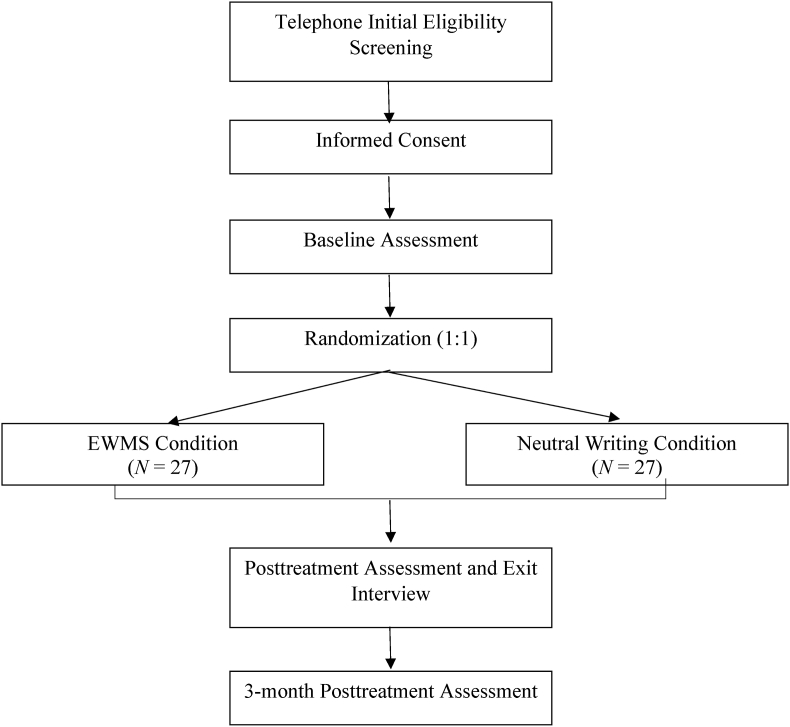
Participant flow diagram.

See Fig. 1 for participant flow diagram. All interested potential participants will contact the research team to complete a phone screening to determine initial eligibility for the study. Initial eligibility will include: 1) being a veteran, 2) identifying as a sexual minority person, 3) experiencing a minority stressor that is contributing to distress, and 4) if on a psychotropic medication, on a stable dose for at least 4 weeks. If initially eligible based on the phone screening, potential participants will complete informed consent and complete the baseline assessment (diagnostic interview via phone and online self-report questionnaires) to determine eligibility for the study.

#### 3.3.3 Measures

**Eligibility.** Following screening and consent, the PHQ-9 and GAD-7 will be used to assess for inclusion criteria (i.e., score above 10 on either). The SCID mood, psychotic, and substance use modules will be administered to assess current psychosis, mania, or severe substance use disorder. Additionally, the C-SSRS will be administered to assess the presence of current suicidal plan and/or intent.

**Feasibility.** Feasibility of the study design will be measured by the rate and number of referrals for the study and the retention rates for participants across study procedures. Level of feasibility of the intervention will be assessed by examining the percentage of treatment completion.

**Acceptability.** Participants will complete the Client Satisfaction Questionnaire (CSQ-8) [41] to assess treatment satisfaction. Study clinicians for the trial will complete the self-report measure assessing attitudes towards the intervention using the Evidence-Based Practice Attitudes Scale-50 anchored to EWMS [42]. The study team will conduct exit interviews with all veterans at the post-treatment assessment and with clinicians towards the end of Phase II. Consistent with treatment development and implementation considerations, interviews will assess veterans' and clinicians' perceptions of the feasibility, acceptability, usefulness and helpfulness of the intervention, cultural relevance and appropriateness of the intervention, as well as any recommendations for modifications to the intervention to improve the fit of the intervention for the patient population and VHA clinical setting [43,44]. Interviews will be audio recorded for transcription purposes and to inform development of the interview guide for the fully powered clinical trial. Some interview questions will be derived deductively by identifying categories at the beginning of the project. These areas will focus on participants’ perceptions of feasibility and acceptability of the EWMS intervention format and content (e.g., length of treatment, writing prompts). Other questions will be derived inductively by identifying emergent themes. Interview transcripts will be analyzed using a rapid content analysis approach widely used when developing and modifying interventions in VHA [45]. Data collected from the interviews in Phase I (open trial) will be used to inform modifications to the intervention protocol that will be tested in Phase II. Qualitative data collected during Phase II will be used to inform modifications to the protocol for future implementation and testing of the intervention.

**Preliminary Outcomes.** The primary preliminary outcomes are depression and anxiety measured with the PHQ-9 and GAD-7, along with the Depression, Anxiety, and Stress Scale [DASS-21] [46]. Although not the primary focus, the mood and anxiety modules of the SCID will be administered at each time point to describe the sample and allow us to determine whether participants meet criteria for depressive and anxiety disorders, in addition to their self-report measures. Regarding stressors, assessments will involve measures that assess trauma history (Life Events Checklist for DSM-5 [LEC-5] [47] and distal minority stressors (Everyday Discrimination Scale [EDS] [38], victimization scale [48]). Additional preliminary outcomes to be examined will include proximal minority stress (Internalized Homophobia Scale-Revised [49]; two-items assessing outness and concealment [50,51]), community connectedness (LGBT Community Scale [52]), substance use quantity and frequency (Quick Drinking Screen [53] and Alcohol, Smoking, and Substance Involvement Screening Test [WHO-ASSIST] [54]), and PTSD symptom severity (PTSD Checklist for DSM-5 [55]).

**Assessments.** Participants will complete assessments at baseline, posttreatment, and 3-month follow-up that entail an online Qualtrics survey and the telephone diagnostic interview with a master's or doctoral level clinician. Independent assessors, who will be Master's or doctoral level clinicians separate from the study therapists, will administer the C-SSRS and the SCID-5 mood and anxiety modules at each time point and the psychotic and substance use disorder modules at baseline. Independent assessors will be trained by a leading expert in assessment for clinical trials and participate in weekly reliability meetings to increase fidelity and reduce rater drift. Interrater reliability will be calculated for a subsample (10%) of the assessments. Assessors will confirm participants' location and will be provided with the participants' current address, emergency contact, and nearest emergency room (collected at consent) to enable required actions if a participant endorses acute suicidal risk and it is determined clinical intervention is required. Participants who are deemed high acute risk at the baseline assessment will not be randomized for treatment and will be connected to emergency resources. Participants will be paid $75 for the baseline assessment and $50 for the posttreatment and 3-month follow-up assessments.

See Table 1 for list of measures and assessment time points.

**Table 1:** Assessment measures and timepoints.

	Time of Administration		
	Baseline	Post-treatment	3-month follow-up
**Screening and Primary Treatment Outcomes**			
SCID-5 modules [40]:			
Mood disorders	X	X	X
Anxiety disorders	X	X	X
Psychotic disorders	X		
Substance use disorders	X		
Columbia Suicide Severity Rating Scale (C-SSRS) [39]	X	X	X
Patient Health Questionnaire (PHQ-9) [36]	X	X	X
Generalized Anxiety Disorder Scale (GAD-7) [37]	X	X	X
**Psychological Measures and Secondary Treatment Outcomes**			
Everyday Discrimination Scale (EDS) [38]	X	X	X
LGBTQ + Victimization [48]	X	X	X
Life Events Checklist (LEC) [47]	X		
PTSD Checklist for DSM-5 (PCL) [55]	X	X	X
Outness & Concealment [50,51]	X	X	X
LGBT Community [52]	X	X	X
Internalized Homophobia Scale (IHS) [49]	X	X	X
Quick Drinking Screen (QDS) [53]	X	X	X
Alcohol, Smoking, and Substance Involvement Screening Test (WHO-ASSIST) [54]	X	X	X
Depression, Anxiety, and Stress Scale (DASS-21) [46]	X	X	X
**Feasibility and Acceptability**			
End-of-treatment interviews		X	
Client Satisfaction Questionnaire (CSQ-8) [41]		X	

### 3.4 Interventions

#### 3.4.1 EWMS

The EWMS protocol will consist of three, 60-min sessions delivered by a clinician (either in-person or via telehealth, based on veteran's preference). The intervention will begin with brief psychoeducation about expressive writing and psychoeducation on sexual minority stressors and common reactions to these stressors. Participants will be provided with a structured prompt for the expressive writing portion of the sessions where they will be asked to write for 30 min about the most upsetting event that has happened to them related to their sexual orientation for the 3 sessions. The writing prompts will be revised throughout the case series portion of the study to ensure cultural relevance to sexual minority veterans. Following the writing exercise, the clinician will check in with the participant about how the writing went and how it felt to write about the chosen stressor.

#### 3.4.2 Control condition

The control writing condition will also be a 3-session individual intervention involving a writing exercise each session. For the control writing exercises, participants will be asked to write for 30 min about their daily activities since waking up that day in a manner that is similar to Pennebaker's neutral writing condition (expressing no thoughts or feelings about the daily activities) [19]. Individuals in the control condition will also be given information about the purpose of the writing exercises to be comparable to the psychoeducation information provided in EWMS. Like EWMS, the clinician will check in with the participant about the writing session, such as asking how the session went and how it felt to complete the writing.

#### 3.4.3 Study clinician training

For Phase II, at least 3 clinicians from mental health clinics at [masked] will be trained in EWMS and the control condition in a half-day training. The PI will provide training and weekly consultation to study clinicians, who will be master's or doctoral level clinicians, operating under licensed oversight from the PI. The PI will review session recordings of each clinician's first case after each session and prior to the next session to ensure compliance with protocol. Additionally, the PI will intermittently review sessions thereafter to ensure fidelity to the protocol is maintained. The PI is a licensed clinical psychologist and will be informed about any clinical worsening, presence of suicidal ideation or adverse events in weekly supervision. The PI will be alerted in situations of high acute risk to help, if needed, in connecting participants to emergency resources.

### 3.5 Power analysis

Consistent with recommendations for pilots, the purpose of the pilot trial is to examine feasibility and acceptability of the study design and intervention to inform the design of a future, fully powered efficacy trial [56,57]. As such, our proposed sample size is consistent with recommendations for pilot trial sample sizes, 12 to 35 per arm [[58], [59], [60], [61], [62]].

### 3.6 Analytic plan

#### 3.6.1 Feasibility and acceptability

We will conduct descriptive statistics and data visualizations to describe the feasibility and acceptability data. We will calculate the rate (referrals per month) and total number of referrals, screens, participants who were eligible, participants who were randomized, and participants who completed the assessments. We will calculate retention rates by examining the number of eligible participants who completed all study procedures. For feasibility of the intervention, we will examine percentage of treatment completion across participants. Regarding acceptability, we will conduct descriptive statistics and data visualizations to describe scores on the CSQ-8 [41] and the EBPAS-50 [42].

To inform necessary modifications to the EWMS protocol, we will conduct rapid content analysis on the exit interviews, which allows for a quick and accurate reduction of qualitative data and produces results useful for efficient intervention refinement [45]. In this approach, data analysis occurs concurrently with data collection. As interviews become available, they will be reviewed and summarized by members of the research team using structured templates organized by key topics from the interview guides and will include information on emergent topics. Analysis will be conducted by a team of 3 at minimum. We will transfer the summaries into matrices and use matrix analysis methods to identify key themes from the interviews. Matrices streamline the process of summarizing data to enable identification of similarities, differences, and trends in responses across interviews, expediting synthesis and summary of findings [63]. As analysis continues, we will identify themes that are evident across multiple interviews. These cross-cutting themes will become the framework for our final analysis, for which we will use a hybrid deductive and inductive analytic approach [45]. We will characterize perceptions of feasibility, acceptability, usefulness, barriers and facilitators to participation, and recommendations for future implementation.

#### 3.6.2 Preliminary outcomes and descriptive trend analyses

Because the trial is a pilot study in preparation for a fully powered clinical trial, we will conduct descriptive statistics and data visualizations to describe the data. We will present the means, standard deviations, and ranges for each of the study time points for the PHQ9, GAD7, and DASS subscales, and produce visual plots to display outcomes by group over time. Lastly, we will examine effect sizes for primary outcomes of interest, including the PHQ-9, GAD-7, and DASS, between groups. Effect sizes (Cohen's *d*) will be calculated as the change from baseline to posttreatment and baseline to 3-month follow-up, standardized by the pooled baseline standard deviations for the EWMS and neutral writing condition. This will indicate the magnitude of change at each posttreatment time point.

## 4 Discussion

This paper provides an overview of an open trial and pilot RCT to test the feasibility and acceptability of a new, brief intervention for sexual minority veterans, EWMS. We describe how data collected in the trial, including interview data from veterans and clinicians, will guide refinements to the EWMS intervention to strengthen the feasibility, acceptability, and usefulness of the intervention. The data from this clinical trial, including the preliminary trends in depressive and anxiety symptom severity, will inform the development of a fully powered efficacy trial for EWMS with sexual minority veterans. To our knowledge, this is the first RCT of an individual psychotherapy intervention for sexual minority veterans. As such, this pilot RCT has the potential to inform not only a fully powered efficacy trial of EWMS, but also other research on intervention adaptation, testing, and implementation for sexual minority veterans.

If EWMS is acceptable and feasible to sexual minority veterans in this trial and then efficacious in a future trial, this intervention may be useful in reducing depression and anxiety among sexual minority veterans. As sexual minority stressors are frequently experienced by sexual minority veterans compared with even their non-veteran peers [4], this intervention has the potential to provide a wide-scale impact in reducing the impact of these experiences. Expressive writing based interventions, including WET for PTSD [64], have been effectively implemented in various outpatient clinic settings, inpatient settings, with telehealth video platforms, and in online, self-directed (i.e., without a therapist) formats [18,30]. Future research should continue to explore the effectiveness of delivering EWMS across a range of settings, including potentially in an online, self-directed format to increase accessibility. Research is needed on the potential use of EWMS in a stepped care or adjunctive approach. For example, for some people EWMS may be effective and sufficient for addressing mental health difficulties. However, for others EWMS may function best as an adjunctive treatment in addition to other interventions or as an entry treatment to address identity-related distress prior to engaging in other treatment.

This pilot trial also incorporates design elements that can help accelerate the timeline between intervention development, refinement, and implementation (i.e., elements of a type 1 effectiveness-implementation trial) [65]**.** There is a lengthy gap between the development and testing of clinical interventions and their implementation into routine care [66]. This delay between research discovery and clinical uptake is slowed by studies engaging in little systematic effort to understand the potential for implementation during early evaluation of interventions. An innovative and efficient solution is to collect implementation data in the context of early treatment development and evaluation research to inform refinements that will facilitate the intervention's ultimate integration into routine care [67]. Therefore, data collected from interviews with both veterans and clinicians will inform future refinements and implementation strategies that can be tested in a fully-developed hybrid effectiveness trial [65].

Lastly, as intervention research for sexual minority veterans is limited in VHA, this trial could help identify useful intervention research practices for sexual minority veterans. Prior work has established recruitment strategies for sexual and gender minority veterans [68,69]; however, these are limited to survey-based and qualitative studies instead of interventional research, which may require additional or different recruitment strategies. As sexual minority veterans may have understandable distrust in participating in research given historical policies in the military and concerns about mistreatment, this trial will also provide data on acceptability of the research design and information on trust and value in participating in research [70].

Conducting this pilot trial is a first step to establishing an evidence base for expressive writing interventions targeting minority stress for sexual minority veterans. Future powered clinical trials should focus on establishing efficacy of EWMS compared with treatment as usual and active controls (e.g., stress management skills) [71,72]. Additionally, trials with a longer follow-up time period will be needed to determine whether any treatment gains are maintained over time [73]. Additional future trials would also benefit from exploring potential mediators and moderators of EWMS. Prior research on expressive-writing interventions have established few mechanisms of change for these interventions [74]; however, key mechanisms that may benefit from examination in future trials are cognitive processing and emotion regulation [75]. Prior research has identified several moderators, including severity of minority stressors and outness [18,33], that would also be useful to explore in future fully-powered trials. Lastly, future research should examine the impact of traumatic event exposure and PTSD on treatment outcomes. In this trial, we will collect information on trauma exposure and PTSD symptom severity, along with examining whether the events participants choose to write on qualify as DSM-5 Criterion A traumatic events; however, we will be underpowered to examine differences in outcomes based on whether individuals write on events that meet this threshold. Future research would benefit from examining the impact of writing on Criterion A bias-related events (e.g., bias-related sexual assault), non-Criterion A bias-related events (e.g., verbal harassment), and Criterion A non-bias related events (e.g., motor vehicle accident, natural disaster) in reducing symptom severity.

This paper provides a description of a pilot RCT to test the feasibility and acceptability of a novel, brief intervention for sexual minority veterans, EWMS. Results from the trial will inform refinements to the intervention, including using implementation data to increase the likelihood of uptake of the intervention in clinical practice, along with also informing a future fully powered trial to test the efficacy of the intervention. As sexual minority stressors and related negative psychological sequalae are highly prevalent among sexual minority veterans, this trial has the potential to inform imperative intervention development, testing, and implementation research to reduce disproportional mental health difficulties among this community.

## Disclaimer statement

The contents do not represent the views of the U.S. Department of Veterans Affairs or the United States Government.

## Relationships

There are no additional relationships to disclose.

## Patents and intellectual property

There are no patents to disclose.

## Other activities

There are no additional activities to disclose.

## Funding

Kelly L. Harper was supported by the 10.13039/100000738U.S. Department of Veterans Affairs (Clinical Sciences Research and Development Service) under Career Development Award # 1IK2 CX002629.

## CRediT authorship contribution statement

**Kelly L. Harper:** Conceptualization, Funding acquisition, Investigation, Methodology, Project administration, Resources, Supervision, Validation, Visualization, Writing – original draft, Writing – review & editing. **Katherine Kelton:** Conceptualization, Methodology, Writing – original draft, Writing – review & editing. **Nicholas A. Livingston:** Conceptualization, Funding acquisition, Methodology, Writing – original draft, Writing – review & editing. **Katherine M. Iverson:** Conceptualization, Funding acquisition, Methodology, Writing – original draft, Writing – review & editing. **Colleen A. Sloan:** Conceptualization, Methodology, Writing – review & editing. **Abigail Batchelder:** Conceptualization, Methodology, Writing – review & editing. **Brian P. Marx:** Conceptualization, Funding acquisition, Methodology, Supervision, Writing – review & editing.

## Declaration of competing interest

The authors declare that they have no known competing financial interests or personal relationships that could have appeared to influence the work reported in this paper.

## Data Availability

No data was used for the research described in the article.
